# αPD-1-mesoCAR-T cells partially inhibit the growth of advanced/refractory ovarian cancer in a patient along with daily apatinib

**DOI:** 10.1136/jitc-2020-001162

**Published:** 2021-02-13

**Authors:** Juemin Fang, Na Ding, Xianling Guo, Yan Sun, Zhiwei Zhang, Bailu Xie, Zhong Li, Hui Wang, Wei Mao, Zhicai Lin, Fei Qin, Min Yuan, Wenqi Chu, Huanlong Qin, Qijun Qian, Qing Xu

**Affiliations:** 1 Department of Medical Oncology, Shanghai Tenth People’s Hospital, Tongji University Cancer Center, School of Medicine, Tongji University, Shanghai 200072, China; 2 Department of Oncology, Shanghai Dermatology Hospital, Tongji University, Shanghai 200072, China; 3 Cell Drug Business Unit, Shanghai Cell Therapy Group Corporation, Shanghai 201805, China; 4 Department of Oncology, Affiliated Hospital of Hebei University of Engineering, Handan 056002, China; 5 Shanghai Cell Therapy Research Institute, Shanghai Mengchao Cancer Hospital, Shanghai University, Shanghai 201805, China

**Keywords:** immunotherapy, receptors, chimeric antigen, case reports, ovarian cancer, immunotherapy, PD-1, Apatinib, CAR-T

## Abstract

Epithelial ovarian cancer (EOC) is the leading cause of death among gynecological malignancies in China. In particular, advanced/refractory ovarian cancer lacks effective targeted therapies due to the immunosuppressive and proangiogenic tumor microenvironment. Mesothelin (MSLN) has been found to be highly expressive in most EOC. Targeting MSLN by antibodies or chimeric antigen receptor-modified T (CAR-T) cells and immune checkpoint blockades as well as apatinib, an anti-angiogenic drug, have been used in patients with refractory ovarian cancer. Apatinib was reported to promote the infiltration of CD8^+^ T cells in lung cancer. However, the combination therapy of CAR-T secreting anti-PD-1 antibody with apatinib in EOC has not been reported.

**Case presentation:**

Here we report a case of refractory EOC in a patient who had relapsed after multiline chemotherapy. The patient received autologous T cells that contained sequences encoding single-chain variable fragments specific for MSLN and full-length antibody for PD-1 (αPD-1). The modified T cells were called αPD-1-mesoCAR-T cells. After infusion, the copy number and PD-1 antibody secretion of the CAR-T cells were increased in the blood. By application of multimodality tumor tracking, MRI of the liver showed shrinkage of metastatic nodules from average diameter of 71.3–39.1 mm at month 2. The patient achieved partial response and survived more than 17 months. IL-6 levels in the patient fluctuated from the baseline to 2–4-folds after treatment, but side effects were mild with only grade 1 hypertension and fatigue.

**Conclusion:**

αPD-1-mesoCAR-T cell therapy combined with apatinib demonstrates a potential therapeutic effect on advanced refractory ovarian cancer.

**Trial registration number:**

NCT03615313.

## Background

Malignant ovarian cancer ranks the second most common cause of gynecologic cancer death in women around the world.^1^ Epithelial ovarian cancer (EOC) accounts for 90% of all ovarian malignancies, and more than 75% of patients are at advanced stages at the time of diagnosis.^2^ Upfront treatment largely relies on debulking surgery and platinum-based chemotherapy with the addition of antiangiogenic agents in patients. Despite aggressive surgery and chemotherapy, most women will ultimately die from the disease.^1^ Therefore, it is necessary to develop new effective therapeutic strategies for patients with advanced or refractory metastatic ovarian cancer.

Chimeric antigen receptor (CAR)-modified T cells (CAR-T cells) have shown promising efficacy in treating hematologic tumors such as relapsed/refractory B-cell leukemia and lymphoma.^3^ However, the response of CAR-T cells, cell therapy in patients with solid tumors is still poor because ideal tumor-specific antigens are rare. Mesothelin (MSLN) is a differentiation antigen with high expression in ovarian cancer but low in normal tissues,^4^ and typically associated with chemoresistance and poor prognosis in advanced EOC.^5^ In our previous study, targeting mesoCAR-T cells significantly suppressed the growth of MSLN-positive ovarian cancer in vitro and in vivo.^6^

Existence of immunosuppressive pathways, especially the PD-1 and PD-L1 axis, can limit the full potential of adoptive T-cell therapy. Blockade of PD-1 by the anti-PD-1 antibody can significantly enhance the antitumor efficacy of CAR-T cells and reverse immunosuppression.^7^ Engineered CAR-T cells to secrete PD-1-blocking single-chain variable fragments (scFv) have been demonstrated to protect CAR-T cells from PD-1 inhibition and enhance antitumor efficacy in preclinical models.^8 9^ To improve the treatment of EOC by CAR-T cells, we generated CAR-T cells with *piggyBac* (PB) transposon vector encoding scFV for MSLN and full-length antibody for PD-1 (αPD-1-mesoCAR-T cells), hopefully to overcome the immunosuppressive tumor microenvironment (TME) and enhance antitumor activity.

Apatinib, as a promising antiangiogenic drug and small-molecule tyrosine kinase inhibitor of vascular endothelial growth factor receptor (VEGFR)-2, has been used in advanced gastric cancer, non-small cell lung cancer, breast cancer and ovarian cancer after multiline therapies.^10 11^ The combination of antiangiogenic agents with immunotherapy has also improved efficacy in solid tumors.^12–14^

In this study, patient with ovarian cancer with a failure history of chemotherapy was given two infusions of αPD-1-mesoCAR-T cells in combination with apatinib. Synergistic inhibition of liver metastatic nodules was observed by MRI. The patient achieved partial response and survived for 17 months and had mild side effects. The results suggest that the combination of CAR-T cells with apatinib would be a new therapeutic way for the treatment of advanced/refractory ovarian cancer.

## Case presentation

### The medical history

**Figure 1:**
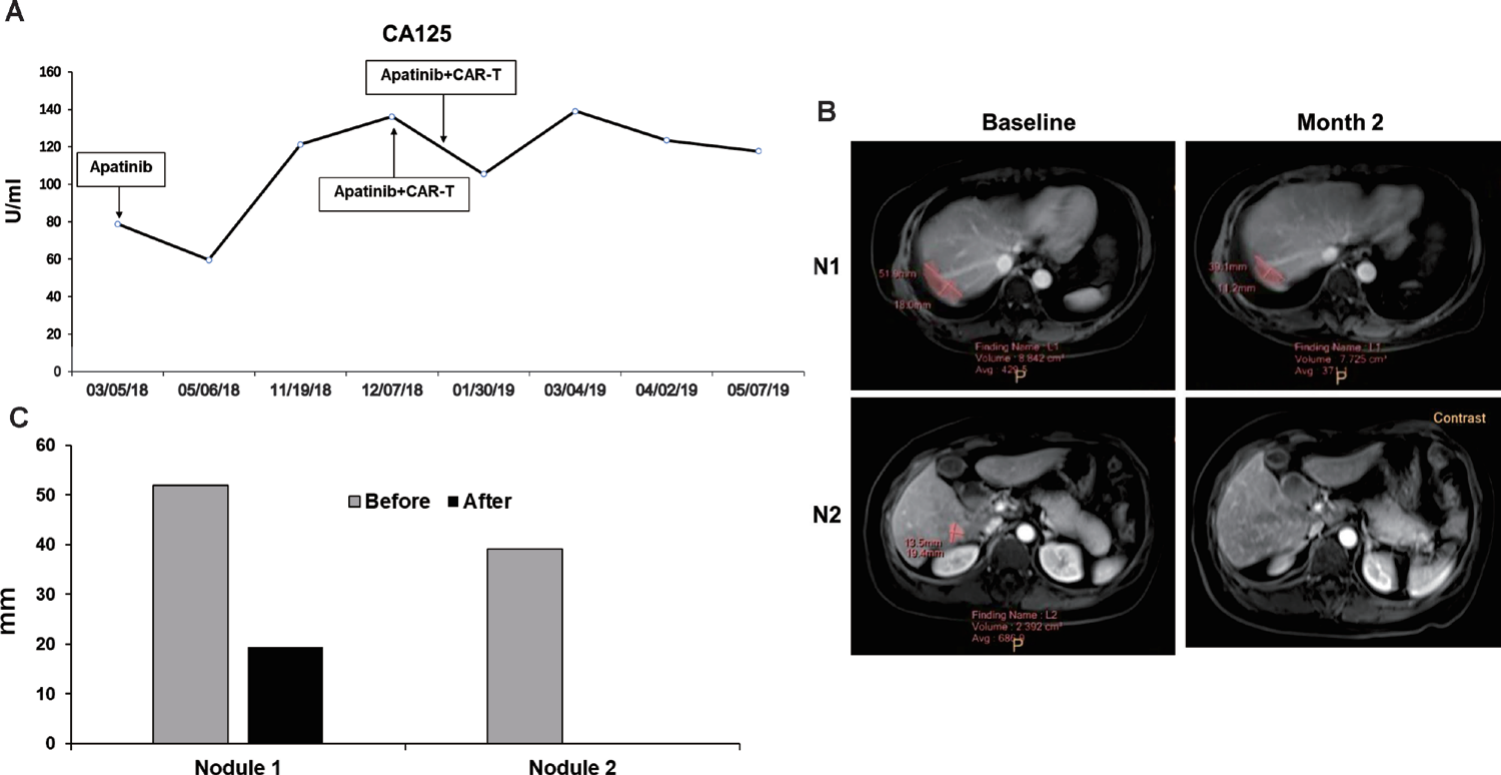
Clinical response. (A) Detection of plasma CA125 levels by Elecsys (Roche) in hospital medical laboratory. The curve starts from the day first taking apatinib to the end of observation period of immunotherapy and shows a decrease at month 2 and an increase at month 8. The two times of CAR-T cell therapies make it down in 2 months of apatinib treatment. (B) Change of the two metastatic lesions (pink areas) before and after immunotherapy in the right hepatic lobe. Upper panels show nodule 1 (N1), lower panels are nodule 2 (N2). (C) Diameters of two metastatic nodules are determined by the multimodality tumor tracking system. The diameter of N1 reduced from 51.9 to 39.1 mm, while N2 from 19.4 mm to undetectable after the combined immunotherapy. CAR-T, chimeric antigen receptor T cells.

**Figure 2:**
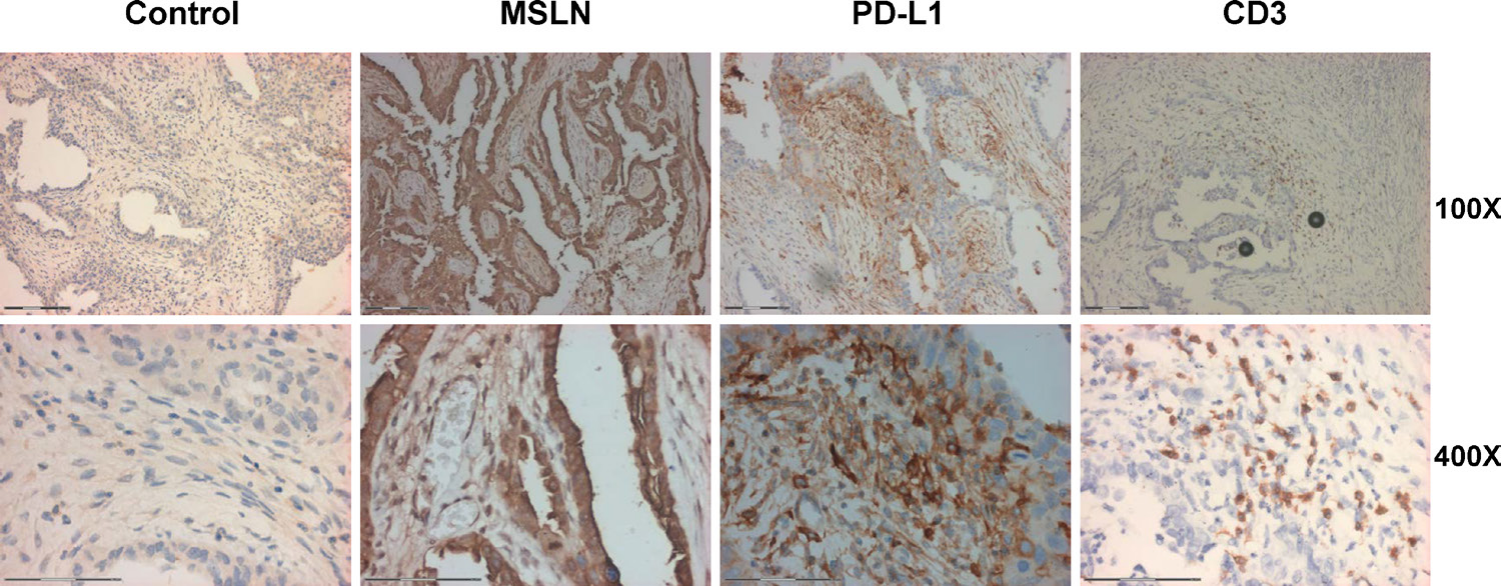
Immunohistochemical analysis of ovarian cancer tissue for MSLN, PD-L1 and CD3 expression. The tissues (5 µm thick) were used for immunohistochemical analysis according to the methods described previously.^15^ There were 84% of tumor cells positive for MSLN staining, 46% of infiltrated lymphocytes were CD3 positive, PD-L1 staining was 35.24%. Magnification of the upper panels are 100×, the scale bar is 150 μm. The low panels are 400×, the scale bar is 74 μm. MSLN, mesothelin.

A 54-year-old woman was initially diagnosed with advanced ovarian serous adenocarcinoma at stage IIIc and had debulking surgery in September 2015. Immunohistochemical staining of the tumor tissue showed positive for CK7(+), CA125(+), WT-1(+), EMA(+), CAM5.2 (+), ER(+), PR(+++), calretinin (partial +), p53(+++), Ki67(60%), CD34(blood vessel +), and negative for Her2, CK20, CA19-9, vimentin, CEA, and HBME-1. The same pathological features were also seen in the staining of left fallopian tube. The patient received firstline combined chemotherapy with paclitaxel plus cisplatin for eight cycles and then second line with gemcitabine plus oxaliplatin for four cycles. Stable disease (SD) was achieved until August 2017 when MRI found new lesions in her liver. Then, liposomal doxorubicin plus nedaplatin was administrated for six cycles. In March 2018, an elevation of CA125 along with the enlargement of the liver lesion occurred and apatinib (250 mg per day, po) was given. CA125 dropped down after treatment (figure 1A) and she was in SD for 8 months. In October 2018, CA125 was elevated. The patient asked for immunotherapy. By confirming, there were two measurable nodules in the liver by MRI (figure 1B), no metastatic lesions were found in the lung and pelvic area (online supplemental figure 1A). Four mismatch repair proteins (MLH1, MSH2, MSH6 and PMS2) were normally expressed and microsatellite was stable (online supplemental figure 1B). Nineteen common genes related with tumorigenesis were also found no mutations (online supplemental tables 1 and 2). However, MSLN(+++84%) staining was strong in her tumor tissue (figure 2), thus the patient was accepted to enroll in the clinical trial of αPD-1-mesoCAR-T cell therapy. The patient signed the informed consent before starting apheresis. The first infusion of CAR-T was initiated in December 2018 and the second one in January 2019. All authors discussed procedures, and interpreted the results and proved the manuscript.

10.1136/jitc-2020-001162.supp4 Supplementary data

10.1136/jitc-2020-001162.supp1 Supplementary data

10.1136/jitc-2020-001162.supp3 Supplementary data

### Autologous CAR-T cell therapy

Peripheral blood mononuclear cells (PBMCs) were collected from the whole blood using a blood cell separator COM.TEC (Fresenius Kabi) according to the manufacturer’s instruction. For the preparation of CAR-T cells, PBMCs were further isolated by Ficoll gradient centrifugation and washed with PBS and suspended in electroporation buffer. Recombinant pNB328-mesoCAR and pNB328-PD1-antibody plasmids (online supplemental figure 1C) were added to the buffer for electroporation. Then, cells were placed in culture medium with MSLN antigen (5 µg/mL) and anti-CD28 (5 µg/mL) and recombinant human IL-2 (200 U/mL) in an incubator at 37°C with 5% CO_2_ for 3 days. After that, the activated cells were continued culturing in the presence of 100 U/mL IL-2 for 14 days. At end of the culture, some of the CAR-T cells were taken for quality control (online supplemental table 3), other cells were collected by centrifugation and suspended again with the cryoprotectant containing 10% dextrn-40, 5% dimethyl sulfoxide, 5% human serum albumin. The suspended cells were aliquoted to 40 mL in the Cryotore bag (Origen Biomedical) placing in the controlled rate CryoMed Freezers 7451 (Thermo Scientific) and then stored in the vapor and liquid phases of liquid nitrogen. At the time of infusion, the frozen bag was transferred to a small liquid nitrogen tank delivering to the hospital. Before infusion, the frozen bag was immediately put into water bath at 37°C and shaken constantly until the cells were completely thawed. The total cells for infusion were taken from the bag according to the bodyweight of the patient and injected into 50 mL of 0.9% NaCl for intravenous drip.

### Clinical findings

This study was approved by the institutional review board of Shanghai Tenth People’s Hospital (IRB No. SHSY-IEC-4.0/18-09/02) and registered at clinical trial.gov. The patient was the first one enrolled in this study in August, 2018 meeting with inclusion criteria, such as failure of multiline chemotherapy, measurable lesions, positive MSLN staining, and so on. Analysis of her tumor tissue had strong MSLN expression (84%), CD3 positive (46%) infiltrating lymphocytes, but PD-L1 expression was relatively weak about 9% (figure 2), By fluorescent multiplex staining, CD3/PD-1 double staining showed an average 0.09% positive, CD8/PD-1 double staining was average 0.13% positive (online supplemental figure 2). These results indicate that the TME is an infiltrated type. We hypothesized that treatment combining with PD-1 antibody and mesoCAR-T cells would have an effect.

10.1136/jitc-2020-001162.supp2 Supplementary data

**Figure 3:**
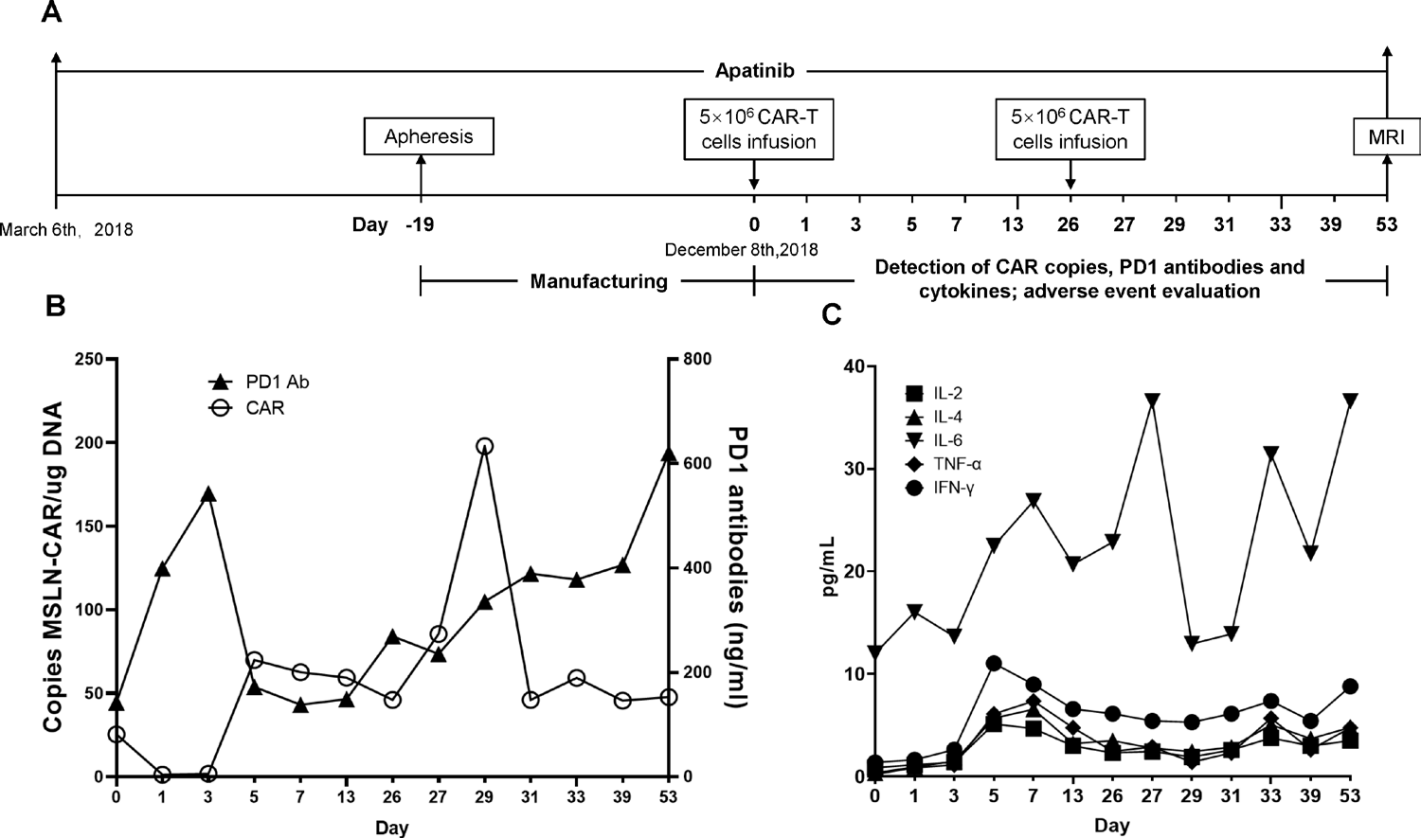
Infusion and expansion of CAR-T cells and levels of PD-1 antibodies and cytokines during treatment. (A) Scheme of apatinib, apheresis, infusion and assessment. (B) Detection of meso-CAR-T and PD-1 antibodies in blood during immunotherapy. Triangles represent PD-1 antibody and circles mean CAR copy number (copies/µg DNA). (C) Cytokine dynamics after two CAR-T infusions (day 0 and day 26). Concentrations of IL-2, 4, 6, tumor necrosis factor (TNF-α) and interferon (IFN-γ) were measured by flow cytometry from day 0 to day 53. Day 0 shows the copy number and PD-1 content of the CAR-T product. CAR-T, chimeric antigen receptor T cells; MSLN, mesothelin.

In this study, the patient received αPD-1-mesoCAR-T cells through intravenous drip on day 0 and day 26 (figure 3A). No lymphodepleting chemotherapy was given before CAR-T cell infusion. The copy number of CAR in the peripheral blood was detected by qPCR as described previously.^15^ In figure 3B, the copy number of the CAR-T product was 25.4 copies/μg DNA showing at day 0. After the first infusion, it was 85.5 copies/μg DNA at day 5, and reached peak level (198 copies/μg DNA) after the second infusion (figure 3B). At day 31, it went down to 46.1 copies/μg DNA. PD-1 antibodies secreted by the CAR-T cells were detected by ELISA. PD-1 antigen proteins were purchased from Abcam (ab174035) and coated on the plate according to the regular protocol. The level of PD-1 antibody was also elevated after infusion and maintained at a constant level, supporting that the αPD-1-mesoCAR-T cells can expand in the body (figure 3B).

The plasma levels of cytokines including IL-2, IL-4, IL-6, tumor necrosis factor (TNF-α) and interferon (IFN-γ) were measured with flow fluorometry during the CAR-T therapy using cytokine detection kit purchased (Saiji Biological Technology). Figure 3C shows that IL-6 increased at day 8 after the first infusion and had two peaks during the second infusion. A high level of IL-6 reflected the activity and proliferation of the CAR-T cells but did not cause severe cytokine releasing syndrome in this patient. Except for IL-6, other cytokines showed a slight increase from day 3 to day 29 of infusion and remained at low levels throughout the study. IFN-γ increased significantly at day 5 after the first infusion and went graduatly donw and kept at higher levels in the period of the treatment. The second infusion had slightly an increase of IFN-γ. Meanwhile, TNF-α, IL-2 and IL-4 had the similar patterns as that of IFN-γ.

Flow cytometry was conducted in the analysis of immune cells before and after the treatment of CAR-T therapy. Total number of leukocytes did not change much after the treatment (online supplemental table 4A), while lymphocyte subsets had changed. CD8^+^ T cells increased to 522 and 612 cells/µl (month 1 and month 3) compared with 352 cells/µl before the treatment, while CD4^+^ T cells decreased to 512 and 522 cells/µl (month 1 and month 3) compared with 800 cells/µl before the CAR-T therapy (online supplemental table 4B). The results suggest that the CAR-T cells may be helpful for enhancing the CD8^+^ T cell activity.

The adverse events (AEs) were evaluated according to the Common Terminology Criteria for Adverse Events (V.5.0). The patient had grade 1 hypertension and fatigue. Other symptoms such as fever, chills, vomiting, and muscle soreness were not observed.

Quantification of segmented lesions was performed using automatic software calculation of selected measurements with long axis by the Multi-Modality Tumor Tracking (Shinefly, Digital China Health, China). The MRI showed that liver lesions shrank after treatment (figure 1B). The diameter of nodule 1 reduced from 51.9 mm to 39.1 mm, while nodule 2 from 19.4 mm to undetectable (figure 1C). Partial response (PR) was achieved according to Response Evaluation Criteria in Solid Tumors V.1.1. The patient had progression-free survival (PFS) for 5 months and survived for 17 months. As shown in figure 1A, slight decline of CA125 was observed after the first infusion of CAR-T cells. Although it rose again after the second infusion, it did not exceed the level prior to the first CAR-T therapy. After the end of 3 months observation, the patient began to receive other therapies.

## Discussion

CAR-T cell immunotherapy has demonstrated high efficacy in hematological malignancy but still lacks evidence of efficacy in solid tumors. In this case, we demonstrated that CAR-T cells targeting MSLN with the ability to secrete PD-1 antibodies with concomitant use of apatinib in chemotherapy-refractory ovarian cancer improve the treatment outcome and prolong PFS, possibly attributed to the synergetic effects of CAR-T cells, PD-1 antibody and antiangiogenesis.

MSLN has been regarded as an attractive target for CAR-T cell therapy because of abundant expression in tumor cells and limited expression in normal cells.^16^ In our previous study, the mesoCAR T cells exhibited rapid and robust killing effects on high MSLN-expressing metastatic ascites-derived pancreatic cancer-1 (ASPC-1） cells and restrained tumor growth in the ASPC-1 xenograft mice model with increased production of IFN-γ.^15^ mesoCAR T cells can effectively inhibit the growth of ovarian tumors in vivo mouse model.^6^

The PD-1 and PD-L1 signal pathway plays a critical role in modulating activation of T lymphocytes and the pathway blockade has elicited durable antitumor responses and long-term remissions in a subset of patients with a broad spectrum of cancer. But response rates vary between 11% and 40% depending on tumor type with PD-1 blockade. One of the reasons for efficacy is attributed to the concentration of PD-1 antibody inside solid tumors. CAR-T cells with the ability to target the antigen on tumor surface can enter to the TME. By taking the advantage of CAR-T cells, we generated CAR-T cells that are not only targeting MSLN positive cancer cells but also secreting PD-1 antibodies to block PD-1 and promote the cytotoxicity of T cells. Consistently, disruption of PD-1 by clustered regularly interspaced shortpalindromic repeats(CRISPR)/Cas9 can overcome the suppressive effect of PD-1 on mesoCAR-T cells and enhance the antitumor effect of mesoCAR-T cells.^17^

Tumor cells can influence the TME by releasing extracellular signals, promoting tumor angiogenesis and inducing peripheral immune tolerance, while the immune cells in the TME are compromised to the growth and evolution of cancerous cells. Apatinib is a novel tyrosine kinase inhibitor that selectively binds and inhibits VEGFR-2^11^ and has shown promising activity in treating ovarian cancer.^18^ Recent studies showed that apatinib is a feasible treatment in patients with recurrent, platinum-resistant EOC^19^ and the synergistic effect of combining antiangiogenesis with immune checkpoint inhibitors.^12^ Zhao *et al* showed that low-dose administration of apatinib with anti-PD-L1 antibody could alleviate hypoxia, increase the infiltration of CD8^+^ T cells and reduce recruitment of tumor-associated macrophages in both mouse models and patients with lung cancer.^13^ Because the metastatic nodules become too small to do a biopsy, we could not determine the effect of aptinib on the TME. Based on the analysis of the patient’s cancer specimen, we found that total PD-1^+^ T cells in the original cancer were 1.5%, CD3^+^/PD-1^+^ T cells were 0.13%, CD8^+^/PD-1^+^ T cells were 0.09%. Although these results would be completely different from metastatic nodules, it could predict that PD-1 antibody secreted from the CAR-T cells would take effect on the PD-1^+^ immune cells. Therefore, the achieved PR in the current case may be considered to be partly attributable to the combination therapy with low dose of apatinib and PD-1 inhibitor secreted from the CAR-T cells.

The AEs of CAR-T therapy in solid tumors were reported much fewer than those in hematologic malignancies treated with CD19-CAR-T.^20 21^ Side effects are the major concern during the immunotherapy and would provide evidence for several issues. A phase 2 study of pembrolizumab revealed that immune-mediated AEs occurred in 22.6% of patients, the most common of that were hypothyroidism (11.2%) and hyperthyroidism (6.6%).^22^ In this study, we postulated that PD-1 antibodies secreted by CAR-T cells would localize where CAR-T cells stayed and expanded. The level of PD-1 antibody in the peripheral blood may be lower than the TME. The mild adverse reactions indicate the feasibility of the αPD-1-mesoCAR-T cell therapy. The results are consistent with the clinical case report published by Carl H. June for the feasibility and safety of mRNA MSLN-targeted CAR-T cells.^23^ PB transposon system is the non-viral vector with economical and easy preparation.^24 25^ PB transposon can constitute the coding frame into chromosomes via a “cut and paste” mechanism by which cells are able to have the ability to express CAR structure and PD-1 antibody. It would be the reason why the secreted PD-1 antibody was still at the high level 1 month after CAR-T infusion.

Taken together, our data indicate that αPD-1-mesoCAR T cell therapy would provide a promising immunotherapeutic approach for advanced refractory ovarian cancer and warrant further clinical trials. The limitations of the study are the measurement of the expansion of CAR-T cells and the concentration of PD-1 antibodies in the shrank tumor tissue. Additionally, more patients are needed to validate the clinical responses observed in the current case.

## Conclusions

This case demonstrated that αPD-1-mesoCAR-T cell therapy is a feasible and promising treatment for advanced refractory ovarian cancer. Combination therapy of apatinib with αPD-1-mesoCAR-T cells would provide a potential and effective therapeutic strategy for advanced refractory ovarian cancer.
