# Supplementary material for: αPD-1-mesoCAR-T cells partially inhibit the growth of advanced/refractory ovarian cancer in a patient along with daily apatinib
Source: J Immunother Cancer. 2021 Feb 13;9(2):e001162. doi: 10.1136/jitc-2020-001162 (PMC7887368; doi:10.1136/jitc-2020-001162)
Supplement: Supplementary data [file jitc-2020-001162supp004.pdf]

Supplementary Figure Legends：

Figure S1: A. Structures of PD-1 antibody expression vector and meso-CAR. The mesothelin antibody sequence was derived from the YP218 antibody, which was originally discovered by the NIH (https://www.nature.com/articles/srep09928; US Patent: US9803022: https://patents.google.com/patent/US9803022). PD-1 antibody sequence was derived from https://www.drugbank.ca/drugs/DB09037 and China patent (CN 102131828 B) by Merck Sharp & Dohme B.V. These two sequences were cloned into the PB transposon vector pNB328-EF1α, respectively. B. Chest CT and pelvic MRI before and after CAR-T treatment. Chest CT did not find there was metastatic lesion but showing right pleural effusion. The effusion did not change after CAR-T treatment. Pelvic MRI also showed no metastatic lesion in this patient. C. Immunohistochemistry of tumor tissues for microsatellite instability (MSI). Detection of four mismatch repair proteins (MLH1, MSH2, MSH6 and PMS2) showed positive, suggesting that DNA mismatch repair (MMR) function is normal.

Figure S2. Mutiplex fluoresence immunostaining for CD3, CD8 and PD-1 expression. Upper panels show single staining, lower panels show double staining. CD3: Yellow 570nm; CD8: Red 650nm; PD-1: Green 540nm. Quantitation of these three colors was shown in the table below figure. Magnification of the panels are 40X, the scale bar is 35μм.
