# Supplementary figures and images for: αPD-1-mesoCAR-T cells partially inhibit the growth of advanced/refractory ovarian cancer in a patient along with daily apatinib
Source: J Immunother Cancer. 2021 Feb 13;9(2):e001162. doi: 10.1136/jitc-2020-001162 (PMC7887368; doi:10.1136/jitc-2020-001162)

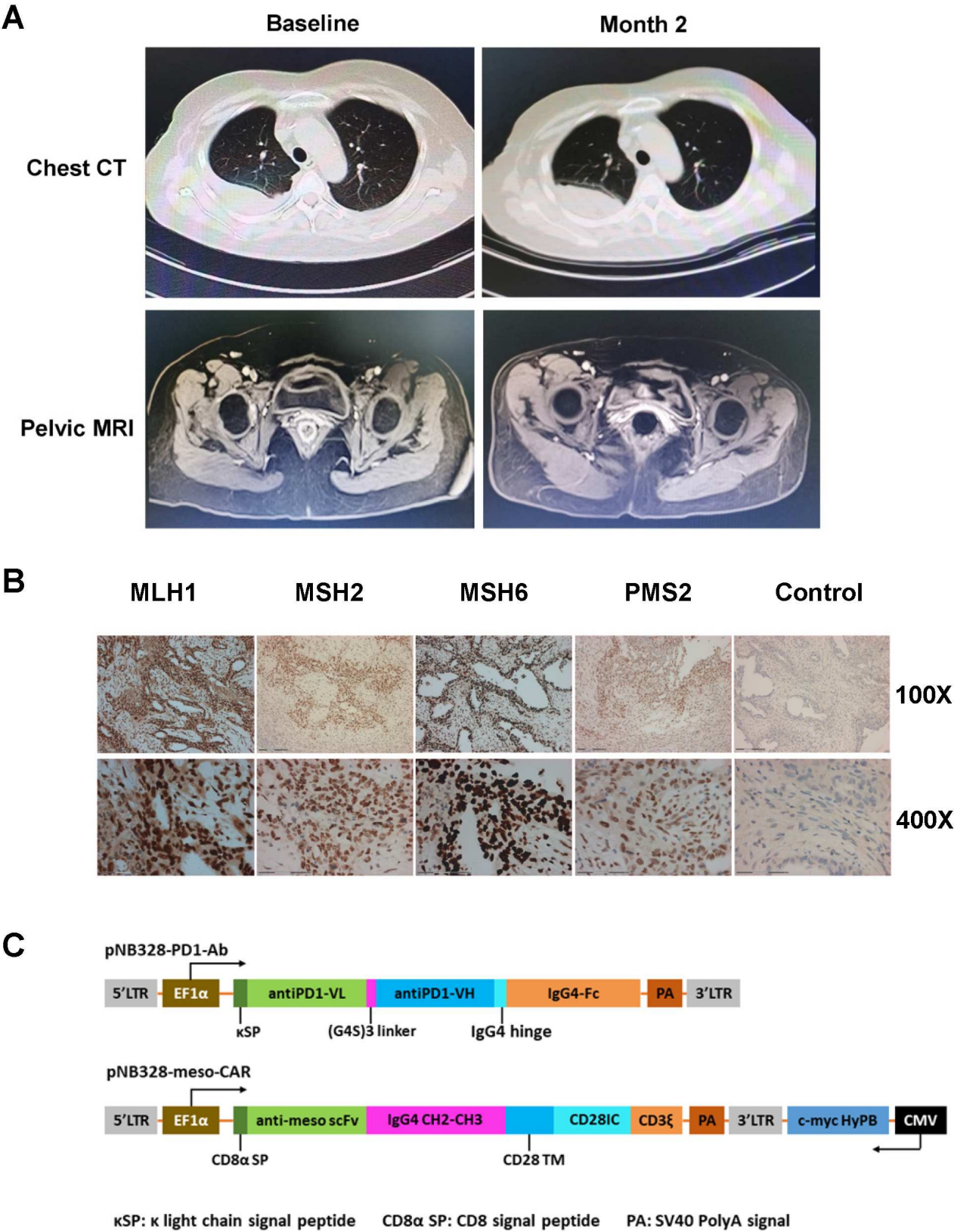

Supplement: Supplementary data [file jitc-2020-001162supp001.pdf]

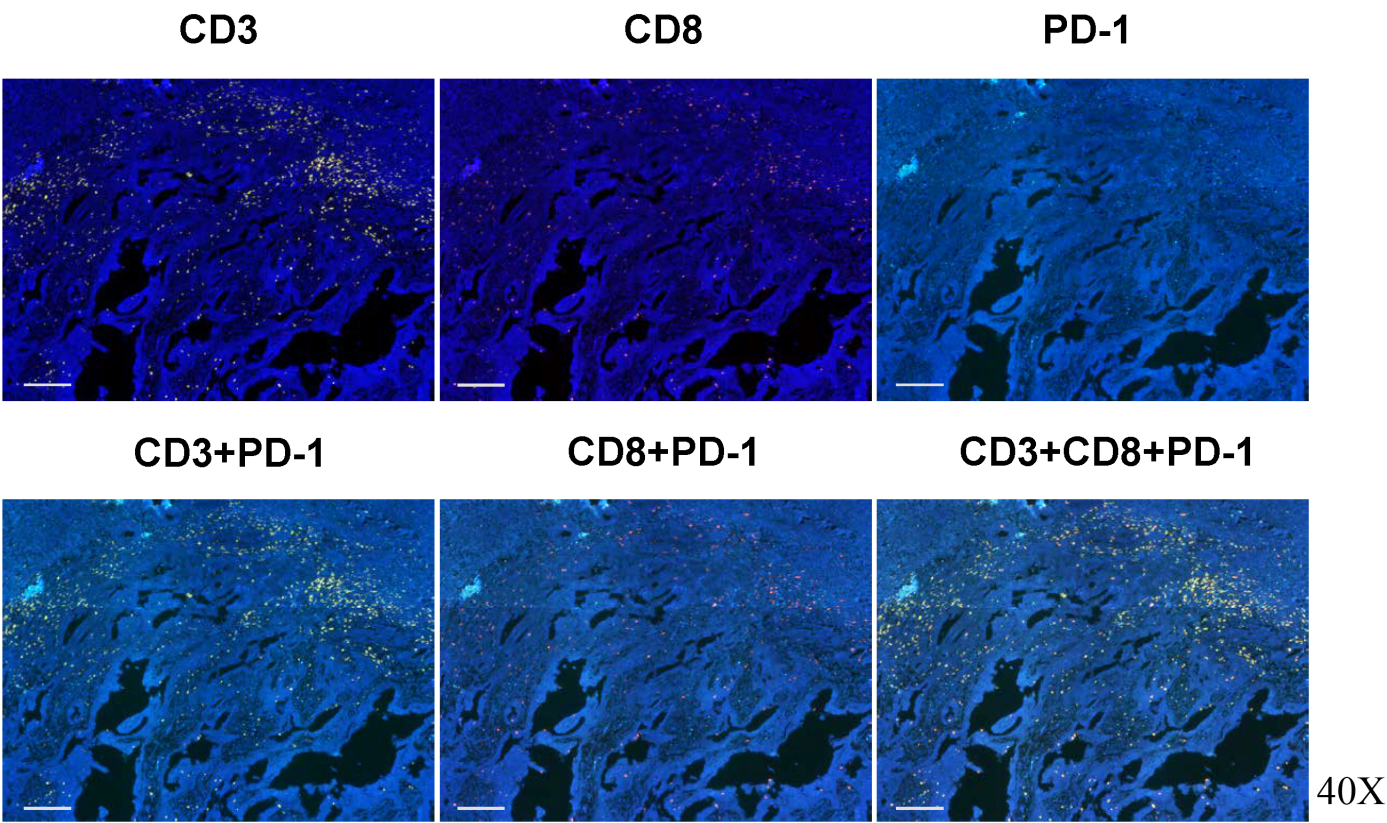

|        |          | positive % |     |      | positive% |      |  | positive% |
|--------|----------|------------|-----|------|-----------|------|--|-----------|
| Single | CD3      | 3.63       | CD8 | 1.67 | PD-1      | 1.57 |  |           |
| Double | CD3+PD-1 | 0.13       | CD3 | 3.55 | PD-1      | 1.51 |  |           |
| Double | CD8+PD-1 | 0.09       | CD8 | 1.54 | PD-1      | 1.44 |  |           |

Supplement: Supplementary data [file jitc-2020-001162supp002.pdf]
