# Supplementary material for: αPD-1-mesoCAR-T cells partially inhibit the growth of advanced/refractory ovarian cancer in a patient along with daily apatinib
Source: J Immunother Cancer. 2021 Feb 13;9(2):e001162. doi: 10.1136/jitc-2020-001162 (PMC7887368; doi:10.1136/jitc-2020-001162)
Supplement: Supplementary data [file jitc-2020-001162supp003.pdf]

Supplemental Table 1. ctDNA sequencing of BRCA1/BRCA2 in patient's tumor specimen

Gene	cDNA changes	Amino acid changes	Mutation abundance
BRCA1	c.2082C>T	p.(=)	50.89
BRCA1	c.134+111C>T	/	50.24
BRCA1	c.4900A>G	p.Ser1634Gly	50.01
BRCA1	c.5215+66G>A	/	48.89
BRCA1	c.2612C>T	p.Pro871Leu	48.45
BRCA1	c.3548A>G	p.Lys1183Arg	48.01
BRCA1	c.3113A>G	p.Glu1038Gly	47.67
BRCA1	c.2311T>C	p.(=)	45.59
BRCA2	c.4563A>G	p.(=)	100
BRCA2	c.-26G>A	/	53.08
BRCA2	c.7806-14T>C	/	50.44
BRCA2	c.7242A>G	p.(=)	49.39
BRCA2	c.3396A>G	p.(=)	49.11
BRCA2	c.3807T>C	p.(=)	48.2

Note: BRCA1, Breast cancer cusceptibility Gene1; BRCA2, Breast cancer susceptibility Gen2.

Supplemental Table 2. ctDNA sequencing of 17 common genes in patient's tumor specimen

Gene	Mutation
AKT1	None
BRAF	None
CTNNB1	None
MLH1	None
MET	None
CDH1	None
ERBB2	None
ERBB4	None
EGFR	None
FGFR1	None
FGFR2	None
FGFR3	None
KRAS	None
PIK3CA	None
PTEN	None
GNAS	None
P53	None

Note:CTNNB1, Catenin Beta 1; MLH1, DNA mismatch repair protein mutL homolog 1; CDH1, Cadherin-1; ERBB2, Receptor tyrosine-protein kinase erbB-2; ERBB4, Receptor tyrosine-protein kinase erbB-2; EGFR, Epidermal growth factor receptor; FGFR, Fibroblast growth factor receptor 1; PIK3CA, Phosphatidylinositol 3-kinase, catalytic subunit alpha.

Supplemental Table 3. Characterization of the CAR-T cells

	Total cells	Cell activity (%)	CD3+ （%）	CD3+MSLN+ （%）	CD3+CD8+ MSLN+ （%）	CD3+CD4+ MSLN+(%)	copies number/ cell	PD-1 antibody (ng/ml)	Bacteria
20181208	0.35x10^8	96.08	70.60	40.63	29.29	10.49	25.70	34.71	negative
20190308	0.57x10^8	90.00	88.56	56.68	34.85	20.65	31.10	40.09	negative

Note: Cell activity was determined by Smart Cell Counter NucleoCounter® NC-200TM (ChemoMetec, Danmark). 200 µL suspended cells was added into Vial-CassetteTM containing dried acridine orange (AO) and DAPI dyes. Dead cells were dyed with AO/PI and counted by NucleoCounter. The results showed as (total cells-dead cells)/ total cells x 100/100. Secreted PD-1 antibody by CAR-T cells was measured by ELISA. PD-1 antigen proteins, purchased from Abcam (ab174035), were dissolved in sterile PBS to a concentration of 0.5µg/ml. 100µl of the antigen solution or PBS was added to 96-well plates for overnight, respectively. After blocking with 1% goat serum for 1 hour at room temperature, 100µl of samples was added to control and test plates. These plates were incubated at 37°C for 45min and then washed five times with PBS. 100µl of substrate solution (tetramethyl benzidine) was added to each well in dark for 10 min and the plates were read at OD450nm by an ELISA reader.

Supplemental Data Table 4. Measurement of leukocyte and lymphocyte subsets before and after the CAR-T treatment

A. Measurement of leukocyte subsets before and after the CAR-T treatment

	White cells (10^9/L)	Monocytes (10^9/L)	Neutrophils (10^9/L)	Lymphocytes (10^9/L)
Before	5.91	0.34	3.9	1.45
After	5.14	0.25	2.9	1.67

B. Measurement of lymphocyte subsets before and after the CAR-T treatment

（Numbers/ul）	Before	Month 1	Month 3
Total T cells	1220	1096	1202
CD8+ T cell	352	522	612
CD4+ T cell	800	512	522
CD4/CD8	2.27	0.98	0.85

Note： Lymphocyte subsets were detected by flow cytometry.
